# (2*Z*)-2-Benzyl­idene-4-*n*-butyl-3,4-di­hydro-2*H*-1,4-benzo­thia­zin-3-one

**DOI:** 10.1107/S160053681401054X

**Published:** 2014-05-21

**Authors:** Nada Kheira Sebbar, Mohammed El Fal, El Mokhtar Essassi, Mohamed Saadi, Lahcen El Ammari

**Affiliations:** a Laboratoire de Chimie Organique Hétérocyclique URAC 21, Pôle de Compétence Pharmacochimie, Av. Ibn Battouta, BP 1014, Faculté des Sciences, Université Mohammed V-Agdal, Rabat, Morocco; b Laboratoire de Chimie du Solide Appliquée, Faculté des Sciences, Université Mohammed V-Agdal, Avenue Ibn Battouta, BP 1014, Rabat, Morocco

## Abstract

In the title compound, C_19_H_19_NOS, the six-membered hetero­cyclic ring of the benzo­thia­zine fragment exhibits a screw boat conformation. The plane of the fused benzene ring makes a dihedral angle of 72.38 (12)° with that of the terminal phenyl ring, and is nearly perpendicular to the mean plane formed by the atoms through the *n*-butyl chain, as indicated by the dihedral angle of 88.1 (2)°. In the crystal, mol­ecules are linked by C—H⋯O inter­actions to form supra­molecular chains along [110].

## Related literature   

For the pharmaceutical and biochemical properties of benzo­thia­zine and their derivatives, see: Malagu *et al.* (1998 ▶); Wammack *et al.* (2002 ▶); Rathore & Kumar (2006 ▶); Zia-ur-Rehman *et al.* (2009 ▶). For related structures, see: Sebbar *et al.* (2014 ▶); Saeed *et al.* (2010 ▶). For puckering calculations, see: Cremer & Pople (1975 ▶).
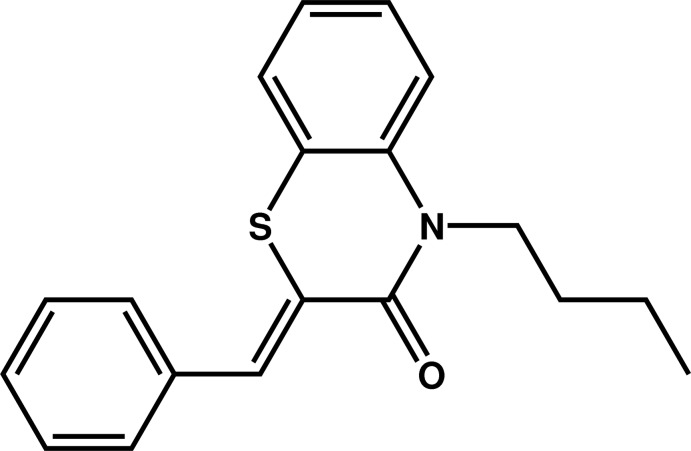


## Experimental   

### 

#### Crystal data  

C_19_H_19_NOS
*M* *_r_* = 309.41
Triclinic, 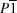

*a* = 8.7717 (13) Å
*b* = 8.8631 (13) Å
*c* = 12.3184 (16) Å
α = 88.283 (9)°
β = 82.302 (9)°
γ = 60.895 (8)°
*V* = 828.5 (2) Å^3^
*Z* = 2
Mo *K*α radiation
μ = 0.20 mm^−1^
*T* = 296 K
0.37 × 0.34 × 0.28 mm

#### Data collection  

Bruker X8 APEX diffractometer
Absorption correction: multi-scan (*SADABS*; Bruker, 2009 ▶) *T*_min_ = 0.641, *T*_max_ = 0.746
17374 measured reflections
3923 independent reflections
2912 reflections with *I* > 2σ(*I*)
*R*_int_ = 0.033

#### Refinement  

*R*[*F* ^2^ > 2σ(*F* ^2^)] = 0.044
*wR*(*F* ^2^) = 0.129
*S* = 1.04
3923 reflections
199 parameters
H-atom parameters constrained
Δρ_max_ = 0.28 e Å^−3^
Δρ_min_ = −0.23 e Å^−3^

### 

Data collection: *APEX2* (Bruker, 2009 ▶); cell refinement: *SAINT-Plus* (Bruker, 2009 ▶); data reduction: *SAINT-Plus*; program(s) used to solve structure: *SHELXS97* (Sheldrick, 2008 ▶); program(s) used to refine structure: *SHELXL97* (Sheldrick, 2008 ▶); molecular graphics: *ORTEP-3 for Windows* (Farrugia, 2012 ▶); software used to prepare material for publication: *PLATON* (Spek, 2009 ▶) and *publCIF* (Westrip, 2010 ▶).

## Supplementary Material

Crystal structure: contains datablock(s) I. DOI: 10.1107/S160053681401054X/tk5313sup1.cif

Structure factors: contains datablock(s) I. DOI: 10.1107/S160053681401054X/tk5313Isup2.hkl

Supporting information file. DOI: 10.1107/S160053681401054X/tk5313Isup3.cml

CCDC reference: 1001815

Additional supporting information:  crystallographic information; 3D view; checkCIF report

## Figures and Tables

**Table 1:** Hydrogen-bond geometry (Å, °)

*D*—H⋯*A*	*D*—H	H⋯*A*	*D*⋯*A*	*D*—H⋯*A*
C4—H4⋯O1^i^	0.93	2.50	3.407 (2)	165

Symmetry code: (i) 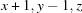.

**Fig. 1.**
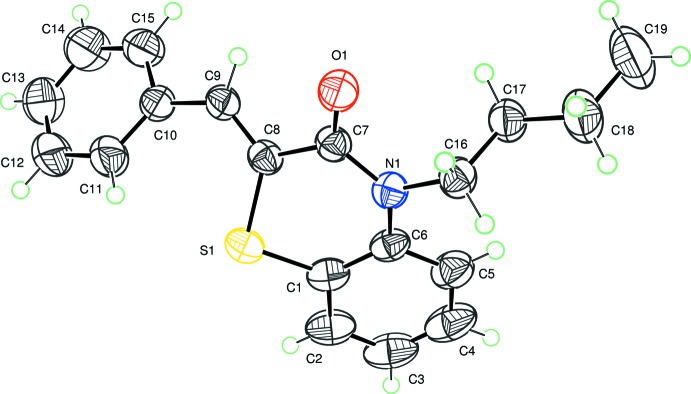
Molecular structure of the title compound with the atom-labelling scheme. Displacement ellipsoids are drawn at the 50% probability level. H atoms are represented as small circles.

**Fig. 2.**
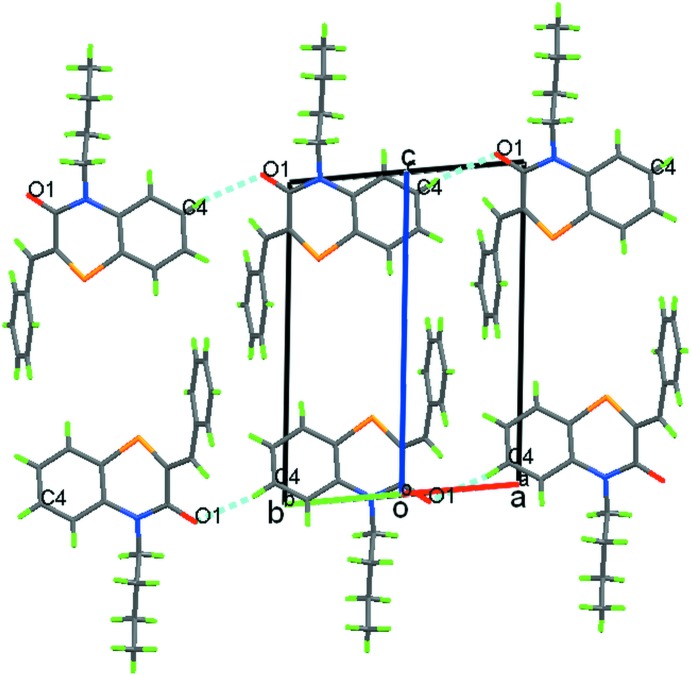
Structure projection along [1 1 0] of the title compound, showing molecules linked through C4–H4···O1 hydrogen bonds (dashed lines).

## supplementary crystallographic information

### 2. Structural commentary

Recently, a number of pharmacological tests revealed that benzo­thia­zine derivatives present various biological activities. These derivatives are found potent analgesic (Wammack *et al.*, 2002); anti-viral (Malagu *et al.*, 1998; Rathore & Kumar, 2006) and anti-oxidant (Zia-ur-Rehman *et al.*, 2009). As a continuation of our research work devoted to the development of *N*-substituted benzo­thia­zine with potential pharmacological activities, we have studied the action of 1-bromo­butane towards 2-(benzyl­idene)-3,4- di­hydro-2*H*-1,4-benzo­thia­zin-3-one under phase transfer catalysis conditions using tetra *n*-butyl ammonium bromide as catalyst and potassium carbonate as base (Saeed *et al.*, 2010; Sebbar *et al.*, 2014) (Scheme 1).

The molecule of the title compound is build up from two fused six-membered rings linked to a phenyl ring and to a *n*-butyl chain as shown in Fig. 1. The benzo­thia­zine fragment adopts a screw boat conformation as indicated by the puckering amplitude Q = 0.4701 (14) Å, and spherical polar angle θ = 70.21 (19)°, with φ = 333.4 (2)° (Cremer & Pople, 1975). The dihedral angle between the plane through the phenyl ring (C9 to C15) and the benzene ring (C1 to C6) is 72.38 (12)°. The mean plane formed by the atoms belonging to the *n*-butyl chain (C16 to C19) is nearly perpendicular to the benzene ring as indicated by the dihedral angle between them of 88.1 (2)°.

In the crystal, the molecules are linked by weak inter­molecular C4–H4···O1 inter­actions, in a fashion to form chains along [1 1 0] (see Fig. 2 and Table 1).

### 5. Synthesis and crystallization

To a solution of 2-(benzyl­idene)-3,4-di­hydro-2*H*-1,4-benzo­thia­zin-3-one (0.2 g, 0.7 mmol), potassium carbonate (0.4 g, 2.9 mmol) and tetra *n*-butyl ammonium bromide (0.024 g, 0.07 mmol) in DMF (15 ml) was added 1-bromo­butane (0.20 ml, 1.89 mmol). Stirring was continued at room temperature for 24 h. The mixture was filtered and the solvent removed. The residue was washed with water. The organic compound was chromatographed on a column of silica gel with ethyl acetate-hexane (1/1) as eluent. Yellow crystals were isolated when the solvent was allowed to evaporate (yield = 48% and M.pt = 363 K).

### 6. Refinement

The H atoms were located in a difference map and treated as riding with C—H = 0.93 Å (aromatic), C—H = 0.97 Å (methyl­ene) and C—H = 0.96 Å (methyl), and with *U*_iso_(H) = 1.2*U*_eq_ (aromatic and methyl­ene) and *U*_iso_(H) = 1.5 *U*_eq_(methyl). The (0 0 1) reflection was omitted owing to poor agreement.

### Crystal data

**Table:**

C_19_H_19_NOS	*Z* = 2
*M**_r_* = 309.41	*F*(000) = 328
Triclinic, *P*1	*D*_x_ = 1.240 Mg m^−^^3^
Hall symbol: -P 1	Melting point: 363 K
*a* = 8.7717 (13) Å	Mo *K*α radiation, λ = 0.71073 Å
*b* = 8.8631 (13) Å	Cell parameters from 3923 reflections
*c* = 12.3184 (16) Å	θ = 2.6–27.9°
α = 88.283 (9)°	µ = 0.20 mm^−^^1^
β = 82.302 (9)°	*T* = 296 K
γ = 60.895 (8)°	Block, yellow
*V* = 828.5 (2) Å^3^	0.37 × 0.34 × 0.28 mm

### Data collection

**Table:**

Bruker X8 APEX diffractometer	3923 independent reflections
Radiation source: fine-focus sealed tube	2912 reflections with *I* > 2σ(*I*)
Graphite monochromator	*R*_int_ = 0.033
φ and ω scans	θ_max_ = 27.9°, θ_min_ = 2.6°
Absorption correction: multi-scan (*SADABS*; Bruker, 2009)	*h* = −11→11
*T*_min_ = 0.641, *T*_max_ = 0.746	*k* = −11→11
17374 measured reflections	*l* = −16→16

### Refinement

**Table:**

Refinement on *F*^2^	Primary atom site location: structure-invariant direct methods
Least-squares matrix: full	Secondary atom site location: difference Fourier map
*R*[*F*^2^ > 2σ(*F*^2^)] = 0.044	Hydrogen site location: difference Fourier map
*wR*(*F*^2^) = 0.129	H-atom parameters constrained
*S* = 1.04	*w* = 1/[σ^2^(*F*_o_^2^) + (0.061*P*)^2^ + 0.1635*P*] where *P* = (*F*_o_^2^ + 2*F*_c_^2^)/3
3923 reflections	(Δ/σ)_max_ = 0.001
199 parameters	Δρ_max_ = 0.28 e Å^−^^3^
0 restraints	Δρ_min_ = −0.23 e Å^−^^3^

### Special details

**Table:**

Geometry. All e.s.d.'s (except the e.s.d. in the dihedral angle between two l.s. planes) are estimated using the full covariance matrix. The cell e.s.d.'s are taken into account individually in the estimation of e.s.d.'s in distances, angles and torsion angles; correlations between e.s.d.'s in cell parameters are only used when they are defined by crystal symmetry. An approximate (isotropic) treatment of cell e.s.d.'s is used for estimating e.s.d.'s involving l.s. planes.
Refinement. Refinement of *F*^2^ against all reflections. The weighted *R*-factor *wR* and goodness of fit *S* are based on *F*^2^, conventional *R*-factors *R* are based on *F*, with *F* set to zero for negative *F*^2^. The threshold expression of *F*^2^ > σ(*F*^2^) is used only for calculating *R*-factors(gt) *etc*. and is not relevant to the choice of reflections for refinement. *R*-factors based on *F*^2^ are statistically about twice as large as those based on *F*, and *R*- factors based on all data will be even larger.

### Fractional atomic coordinates and isotropic or equivalent isotropic displacement parameters (Å^2^)

**Table:**

	*x*	*y*	*z*	*U*_iso_*/*U*_eq_	
C1	0.9210 (2)	0.3479 (2)	0.82655 (15)	0.0483 (4)	
C2	1.0667 (3)	0.2113 (2)	0.76691 (19)	0.0648 (5)	
H2	1.0839	0.2136	0.6909	0.078*	
C3	1.1845 (3)	0.0746 (3)	0.8185 (2)	0.0758 (7)	
H3	1.2794	−0.0181	0.7779	0.091*	
C4	1.1622 (3)	0.0746 (3)	0.9299 (2)	0.0761 (7)	
H4	1.2449	−0.0169	0.9653	0.091*	
C5	1.0181 (3)	0.2089 (2)	0.99169 (18)	0.0622 (5)	
H5	1.0048	0.2067	1.0678	0.075*	
C6	0.8931 (2)	0.3472 (2)	0.93992 (14)	0.0447 (4)	
C7	0.6448 (2)	0.6452 (2)	0.96790 (12)	0.0416 (3)	
C8	0.69106 (19)	0.6857 (2)	0.85347 (12)	0.0408 (3)	
C9	0.6528 (2)	0.8484 (2)	0.83178 (12)	0.0449 (4)	
H9	0.5991	0.9273	0.8912	0.054*	
C10	0.6840 (2)	0.9191 (2)	0.72724 (13)	0.0466 (4)	
C11	0.6707 (3)	0.8635 (3)	0.62735 (15)	0.0647 (5)	
H11	0.6374	0.7790	0.6247	0.078*	
C12	0.7061 (3)	0.9318 (3)	0.53127 (16)	0.0779 (7)	
H12	0.6960	0.8930	0.4646	0.093*	
C13	0.7560 (3)	1.0563 (3)	0.53309 (18)	0.0795 (7)	
H13	0.7860	1.0976	0.4680	0.095*	
C14	0.7610 (4)	1.1182 (3)	0.6313 (2)	0.0900 (8)	
H14	0.7893	1.2064	0.6335	0.108*	
C15	0.7245 (3)	1.0520 (3)	0.72774 (17)	0.0720 (6)	
H15	0.7271	1.0971	0.7942	0.086*	
C16	0.6813 (2)	0.4450 (2)	1.11277 (13)	0.0498 (4)	
H16A	0.7185	0.3226	1.1158	0.060*	
H16B	0.5536	0.5075	1.1257	0.060*	
C17	0.7506 (3)	0.4944 (3)	1.20408 (14)	0.0575 (5)	
H17A	0.7010	0.6189	1.2084	0.069*	
H17B	0.8776	0.4429	1.1879	0.069*	
C18	0.7032 (4)	0.4335 (4)	1.31329 (17)	0.0835 (7)	
H18A	0.5767	0.4786	1.3261	0.100*	
H18B	0.7586	0.3084	1.3093	0.100*	
C19	0.7580 (5)	0.4878 (5)	1.4086 (2)	0.1144 (11)	
H19A	0.7221	0.4471	1.4750	0.172*	
H19B	0.7032	0.6116	1.4136	0.172*	
H19C	0.8837	0.4394	1.3983	0.172*	
N1	0.73979 (17)	0.48044 (16)	1.00182 (10)	0.0425 (3)	
O1	0.52331 (16)	0.75693 (15)	1.02912 (9)	0.0587 (3)	
S1	0.77675 (6)	0.51831 (6)	0.75323 (3)	0.05429 (16)	

### Atomic displacement parameters (Å^2^)

**Table:**

	*U*^11^	*U*^22^	*U*^33^	*U*^12^	*U*^13^	*U*^23^
C1	0.0409 (8)	0.0423 (8)	0.0613 (10)	−0.0214 (7)	0.0011 (7)	−0.0073 (7)
C2	0.0519 (11)	0.0527 (10)	0.0814 (13)	−0.0224 (9)	0.0096 (10)	−0.0181 (10)
C3	0.0487 (11)	0.0488 (11)	0.114 (2)	−0.0139 (9)	0.0033 (12)	−0.0223 (12)
C4	0.0515 (11)	0.0422 (10)	0.127 (2)	−0.0129 (9)	−0.0271 (13)	0.0001 (11)
C5	0.0576 (11)	0.0471 (10)	0.0800 (13)	−0.0209 (9)	−0.0224 (10)	0.0038 (9)
C6	0.0390 (8)	0.0371 (8)	0.0612 (10)	−0.0204 (7)	−0.0090 (7)	−0.0005 (7)
C7	0.0403 (8)	0.0431 (8)	0.0394 (8)	−0.0188 (7)	−0.0045 (6)	−0.0009 (6)
C8	0.0358 (7)	0.0442 (8)	0.0376 (7)	−0.0160 (6)	−0.0026 (6)	−0.0032 (6)
C9	0.0464 (9)	0.0444 (8)	0.0371 (7)	−0.0174 (7)	−0.0028 (6)	−0.0013 (6)
C10	0.0470 (9)	0.0444 (8)	0.0422 (8)	−0.0184 (7)	−0.0021 (7)	0.0014 (7)
C11	0.0884 (14)	0.0673 (12)	0.0484 (10)	−0.0453 (11)	−0.0118 (10)	0.0071 (9)
C12	0.1082 (18)	0.0814 (15)	0.0432 (10)	−0.0467 (14)	−0.0055 (11)	0.0038 (10)
C13	0.1030 (18)	0.0853 (16)	0.0524 (12)	−0.0518 (14)	0.0037 (11)	0.0152 (11)
C14	0.139 (2)	0.0867 (16)	0.0709 (15)	−0.0779 (17)	−0.0069 (15)	0.0133 (12)
C15	0.1083 (17)	0.0646 (12)	0.0529 (11)	−0.0508 (12)	−0.0056 (11)	0.0009 (9)
C16	0.0588 (10)	0.0537 (10)	0.0468 (9)	−0.0350 (9)	−0.0085 (8)	0.0085 (7)
C17	0.0750 (13)	0.0627 (11)	0.0488 (9)	−0.0431 (10)	−0.0157 (9)	0.0101 (8)
C18	0.128 (2)	0.1015 (18)	0.0516 (11)	−0.0780 (17)	−0.0226 (12)	0.0191 (11)
C19	0.181 (3)	0.155 (3)	0.0538 (13)	−0.114 (3)	−0.0322 (17)	0.0187 (16)
N1	0.0422 (7)	0.0427 (7)	0.0425 (7)	−0.0207 (6)	−0.0057 (5)	0.0029 (5)
O1	0.0574 (7)	0.0498 (7)	0.0433 (6)	−0.0096 (6)	0.0075 (5)	−0.0014 (5)
S1	0.0622 (3)	0.0504 (3)	0.0421 (2)	−0.0221 (2)	−0.00101 (19)	−0.00837 (17)

### Geometric parameters (Å, º)

**Table:**

C1—C6	1.386 (2)	C11—H11	0.9300
C1—C2	1.391 (2)	C12—C13	1.373 (4)
C1—S1	1.7505 (19)	C12—H12	0.9300
C2—C3	1.361 (3)	C13—C14	1.358 (3)
C2—H2	0.9300	C13—H13	0.9300
C3—C4	1.360 (4)	C14—C15	1.377 (3)
C3—H3	0.9300	C14—H14	0.9300
C4—C5	1.388 (3)	C15—H15	0.9300
C4—H4	0.9300	C16—N1	1.474 (2)
C5—C6	1.394 (2)	C16—C17	1.516 (2)
C5—H5	0.9300	C16—H16A	0.9700
C6—N1	1.421 (2)	C16—H16B	0.9700
C7—O1	1.2199 (18)	C17—C18	1.516 (3)
C7—N1	1.369 (2)	C17—H17A	0.9700
C7—C8	1.496 (2)	C17—H17B	0.9700
C8—C9	1.339 (2)	C18—C19	1.500 (3)
C8—S1	1.7514 (16)	C18—H18A	0.9700
C9—C10	1.466 (2)	C18—H18B	0.9700
C9—H9	0.9300	C19—H19A	0.9600
C10—C11	1.379 (3)	C19—H19B	0.9600
C10—C15	1.386 (3)	C19—H19C	0.9600
C11—C12	1.380 (3)		
			
C6—C1—C2	120.52 (18)	C14—C13—H13	120.5
C6—C1—S1	121.86 (12)	C12—C13—H13	120.5
C2—C1—S1	117.62 (16)	C13—C14—C15	120.8 (2)
C3—C2—C1	120.8 (2)	C13—C14—H14	119.6
C3—C2—H2	119.6	C15—C14—H14	119.6
C1—C2—H2	119.6	C14—C15—C10	121.0 (2)
C4—C3—C2	119.40 (19)	C14—C15—H15	119.5
C4—C3—H3	120.3	C10—C15—H15	119.5
C2—C3—H3	120.3	N1—C16—C17	114.51 (14)
C3—C4—C5	121.1 (2)	N1—C16—H16A	108.6
C3—C4—H4	119.5	C17—C16—H16A	108.6
C5—C4—H4	119.5	N1—C16—H16B	108.6
C4—C5—C6	120.2 (2)	C17—C16—H16B	108.6
C4—C5—H5	119.9	H16A—C16—H16B	107.6
C6—C5—H5	119.9	C16—C17—C18	110.99 (16)
C1—C6—C5	117.94 (16)	C16—C17—H17A	109.4
C1—C6—N1	121.31 (14)	C18—C17—H17A	109.4
C5—C6—N1	120.73 (16)	C16—C17—H17B	109.4
O1—C7—N1	120.87 (14)	C18—C17—H17B	109.4
O1—C7—C8	120.34 (14)	H17A—C17—H17B	108.0
N1—C7—C8	118.79 (13)	C19—C18—C17	113.8 (2)
C9—C8—C7	118.94 (14)	C19—C18—H18A	108.8
C9—C8—S1	123.81 (12)	C17—C18—H18A	108.8
C7—C8—S1	117.05 (12)	C19—C18—H18B	108.8
C8—C9—C10	128.90 (15)	C17—C18—H18B	108.8
C8—C9—H9	115.5	H18A—C18—H18B	107.7
C10—C9—H9	115.5	C18—C19—H19A	109.5
C11—C10—C15	117.53 (17)	C18—C19—H19B	109.5
C11—C10—C9	123.43 (17)	H19A—C19—H19B	109.5
C15—C10—C9	119.02 (16)	C18—C19—H19C	109.5
C10—C11—C12	120.8 (2)	H19A—C19—H19C	109.5
C10—C11—H11	119.6	H19B—C19—H19C	109.5
C12—C11—H11	119.6	C7—N1—C6	124.49 (13)
C13—C12—C11	120.6 (2)	C7—N1—C16	116.46 (13)
C13—C12—H12	119.7	C6—N1—C16	118.97 (13)
C11—C12—H12	119.7	C1—S1—C8	99.56 (8)
C14—C13—C12	119.0 (2)		

### Hydrogen-bond geometry (Å, º)

**Table:**

*D*—H···*A*	*D*—H	H···*A*	*D*···*A*	*D*—H···*A*
C4—H4···O1^i^	0.93	2.50	3.407 (2)	165

Symmetry code: (i) *x*+1, *y*−1, *z*.
